# Antioxidant and Anti-Inflammatory Effects of Coenzyme Q10 on L-Arginine-Induced Acute Pancreatitis in Rat

**DOI:** 10.1155/2016/5818479

**Published:** 2016-04-12

**Authors:** Seyed Abbas Mirmalek, Ala Gholamrezaei Boushehrinejad, Hassan Yavari, Bahareh Kardeh, Yekta Parsa, Seyed Alireza Salimi-Tabatabaee, Soheila Yadollah-Damavandi, Tina Parsa, Ehsan Shahverdi, Ehsan Jangholi

**Affiliations:** ^1^Department of Surgery, Islamic Azad University, Tehran Medical Sciences Branch, Tehran, Iran; ^2^Students' Research Committee, Islamic Azad University, Tehran Medical Sciences Branch, Tehran, Iran; ^3^Young Researchers and Elite Club, Islamic Azad University, Tehran Medical Sciences Branch, Tehran, Iran; ^4^Student Research Committee, Shiraz University of Medical Sciences, Shiraz, Iran; ^5^Students' Research Committee, Baqiyatallah University of Medical Sciences, Tehran, Iran

## Abstract

This study was aimed at evaluating the protective effect of coenzyme Q10 on L-arginine-induced acute pancreatitis in rats regarding biomarkers and morphologic changes. Thirty-two male Sprague-Dawley rats were divided into 4 equal groups. Control group received intraperitoneal normal saline, while in sham and experimental groups 1 and 2 pancreatitis was induced with L-arginine. E1 and E2 groups were treated with a single dose of 100 and 200 mg/kg Q10, respectively. Serum lipase and amylase, along with pancreas IL-10, IL-1*β*, and TNF-*α*, were measured. For evaluation of oxidative stress, pancreatic superoxide dismutase (SOD), glutathione (GSH), malondialdehyde (MDA), and myeloperoxidase (MPO) were assessed. Histopathological examination for morphologic investigation was conducted. Serum amylase and lipase, as well as TNF-*α* and IL-1*β* cytokines, reverted with administration of Q10 in consistence with dosage. In contrast, Q10 assisted in boosting of IL-10 with higher dosage (200 mg/kg). A similar pattern for oxidative stress markers was noticed. Both MDA and MPO levels declined with increased dosage, contrary to elevation of SOD and GSH. Histopathology was in favor of protective effects of Q10. Our findings proved the amelioration of pancreatic injury by Q10, which suggest the anti-inflammatory and antioxidant property of Q10 and its potential therapeutic role.

## 1. Introduction

Acute pancreatitis (AP), noninfectious inflammatory disorder of pancreas, not only is the most common cause of hospital admission among gastrointestinal diseases in many countries [1, 2], but also is recognized as one of the leading acute diseases worldwide, with rising absolute incidence and age-standardized rates over the past decades [3]. Although it generally manifests a self-limited course [4], up to 20% of patients may encounter complications and mortality [5]. Pathogenesis of AP is based on the intracellular autodigestive process, which triggers local and systemic inflammatory response via release of mediators from neutrophils and macrophages in parenchyma. These biochemical interactions eventually lead to acinar injury, interstitial edema, tissue compromise, and systemic presentations blamed for morbidity and mortality in AP [1, 4, 6, 7]. In the inflammation pathways, a number of pro- and anti-inflammatory mediators are activated and diagnosis can be based upon serum level of various chemical biomarkers.

Oxidative stress is one of the pivotal mechanisms of AP. Excessive reactive oxygen species (ROS) provoke inflammation and development of pancreatitis through zymogen degranulation, granulocyte migration, tissue necrosis, and increased amylase and lipase activity [7, 8]. In fact, ROS, as products of oxidation and peroxidation metabolism, are instantly detoxified by natural scavengers and antioxidant agents under normal conditions [9, 10]. In AP, overproduction of ROS and the impaired neutralization ability of scavengers result in ROS accumulation in pancreatic tissue [11]. Therefore, a great therapeutic potential can be acknowledged in enhancement of scavenger defense by utilizing antioxidant agents.

Coenzyme Q10 (Ubiquinone), as the only endogenous lipid soluble antioxidant, with a 2,3-dimethoxy-5-methylbenzoquinone nucleus is well established for its antioxidant and anti-inflammatory effects and is the most frequent type of CoQ in human tissue [12]. Its fundamental roles include energy conversion, antioxidant activity and antioxidant regenerator, cell growth stimulation, and cell death inhibition [12, 13]. It is located proximal to unsaturated lipids and acts as an essential ROS scavenger [13, 14]. In ageing or diseases, CoQ is low and supplementary CoQ can help increase tissue amount in order to reach saturation level; however, except for liver and spleen, it does not rise above the normal [14]. With regard to the central role of ROS in aggravation of AP and mentioned characteristics of Q10, we designed an experimental study to investigate the effects of this enzyme on pancreas histopathological outcomes, as well as changes in serum lipase, amylase, TNF-*α*, IL-1*β*, IL-10, superoxide dismutase (SOD), malondialdehyde (MDA), myeloperoxidase (MPO), and glutathione peroxidase (GSH) in L-arginine-induced AP in rats.

## 2. Materials and Methods

### 2.1. Animal

Thirty-two male Sprague-Dawley rats, weighing 180–200 g, were obtained from the Pasteur Institute, Tehran, Iran. Rats were housed individually in cages on a 12:12 h light-dark cycle at 23 ± 2°C under standard environmental conditions and had ad libitum access to pellet diet and tap water. All of them were housed in animal house for a week prior to experiments. All procedures were performed according to the Guide for the Care and Use of Laboratory Animals (NIH publication number 86-23, 1985 edition) approved by the ethics committee of Islamic Azad University, Tehran Medical Sciences Branch, Tehran, Iran.

### 2.2. Study Design

Rats were randomly divided into 4 groups (*n* = 8). Control group received intraperitoneal (IP) injections of normal saline. In sham and experimental groups, pancreatitis was induced with 3.2 g/kg bodyweight L-arginine (Sigma-Aldrich, Germany) IP, twice at an interval of 1 hour. Rats in E1 and E2 groups were, respectively, treated with a single dose of 100 and 200 mg/kg bodyweight Q10 (Sigma-Aldrich, Germany) IP, 30 minutes prior to L-arginine administration.

### 2.3. Sample Collection

All rats were sacrificed with an overdose of pentobarbital 24 h after the last injection of L-arginine. Blood samples were obtained by direct intracardiac puncture and stored at −70°C for biochemical analysis. The pancreas (4 rats per group) was quickly removed and fixed in formaldehyde (10%) for histological examination.

### 2.4. Measurement of Serum Amylase and Lipase Level

Blood samples were centrifuged at 15,000 rpm under 4°C and the plasma was separated by using sterile pipettes. Serum lipase and amylase activity were evaluated with a spectrophotometric technique by the Olympus AU-2700 autoanalyzer (Olympus, Hamburg, Germany) using commercial kits (MAN Company, Tehran, Iran). The results were expressed as U/I.

### 2.5. Determination of Serum Cytokines

Serum IL-10, IL-1*β*, and TNF-*α* levels were measured using an enzyme-linked immunosorbent assay (ELISA). The blood sample of each group was centrifuged at 3500 r min^−1^ for 15 min. The supernatant was obtained for the analysis of cytokines. These cytokines were measured with ELISA kits (Boster Biological Technology, Wuhan, China) according to the manufacturer's protocol. The ELISA microplate was read using an ELISA reader (Dynatech Laboratories, USA) with an absorbance maximum at 450 nm. The cytokine levels were calculated after plotting the standard curves and expressed as pg/mL.

### 2.6. Evaluation of Oxidative Stress

The pancreatic tissues (4 rats per group) were removed and were promptly frozen in liquid nitrogen and stored at −70°C until being assayed. Protein estimation was done by the method of Lowry et al. [15].

#### 2.6.1. Pancreatic SOD Activity

The activity of SOD in pancreas was measured using a commercial assay kit (Sigma, Germany), following the manufacturer's instructions. This assay kit uses a tetrazolium salt for detection of superoxide anions generated by xanthine oxidase and hypoxanthine. These superoxide radicals oxidize hydroxylamine and lead to formation of nitrite, which reacts with naphthalene diamine and sulfanilic acid to produce a colored product. SOD in the sample reduces the overall superoxide anion concentration, thereby lowering the colorimetric signal and absorbance at 550 nm. One unit (U) of SOD was defined as the amount of enzyme needed to produce 50% dismutation of superoxide radical. The activity of SOD was expressed as U/mg of protein.

#### 2.6.2. Pancreatic GSH Content

The GSH content was measured using the 5,5′-dithiobis(2-nitrobenzoic acid)-oxidized GSH (DTNB-GSSG) reductase recycling assay for total glutathione (GSH + GSSG) as described by Tietze [16]. Briefly, tissues were lysed by 200 *μ*L of lysis buffer (50 mM Tris-HCl, 1 mM EGTA, and 1% Triton X-257 100). The tissue lysate was deproteinized with the same volume of 10% 5-sulfosalicylic acid. After centrifugation at 5000 g for 5 min at 4°C, the supernatant was divided into 2 samples, 1 for GSH and 1 for GSSG measures. The amount of total GSH was determined by formation of 5-thio-2-nitrobenzoic acid converted from DTNB. GSSG was measured by the DTNB-GSSG reductase recycling assay after treating GSH with 2-vinylpyridine for 1 h at room temperature. Total glutathione and GSSG levels were defined as the change in OD at 405 nm for 5 min at room temperature. The results were expressed as *μ*mol/g.

#### 2.6.3. Pancreatic MDA Content

The MDA content was determined using the thiobarbituric acid (TBA) test [17]. In brief, samples were homogenized in 10 mL of TCA (7.5%)-EDTA (0.1%) solution. This sample was shaken continuously for 0.5 h with a mechanical shaker and then filtered. Exactly 5 mL of filtrate was added to 5 mL of TBA (2.88 g/L) solution in a 25 mL colorimetrical tube and heated in a water bath (90°C) for 40 min for pink color development. The tube was first cooled for 1 h and was then centrifuged for 5 min at 3000 g. The supernatant fluid was added to 5 mL of chloroform in another tube and then shaken. This mixed solution was allowed to stand for at least 1 h. The absorbance was measured at 532 nm using a spectrophotometer (UV-2550, Shimadzu, Kyoto, Japan). The results were expressed as nmol/g of protein.

#### 2.6.4. Pancreatic MPO Activity

The pancreatic MPO activity was determined as described [18]. Tissue samples were homogenized in 50 mM potassium phosphate buffer (PB, pH 6.0) and centrifuged at 41, 400 g (10 min); pellets were suspended in 50 mM PB containing 0.5% hexadecyltrimethylammonium bromide (HETAB). After three freeze and thaw cycles, with sonication between cycles, the samples were centrifuged at 41, 400 g for 10 min. Aliquots (0.3 mL) were added to 2.3 mL of reaction mixture containing 50 mM PB, o-dianisidine, and 20 mM H_2_O_2_ solution. One unit of enzyme activity was defined as the amount of MPO present that caused a change in absorbance measured at 460 nm for 3 min. MPO activity was expressed as U/g protein.

### 2.7. Histopathological Examination

Paraffin-embedded pancreas tissues were sectioned (5 *μ*m) and stained with hematoxylin and eosin (H&E). Histopathological changes of the pancreas were evaluated according to a scoring system previously described [19]. The severity of acute pancreatitis was blindly graded by a semiquantitative assessment of edema, inflammatory cell infiltrate, and acinar necrosis, based on the following criteria: (1) edema: 0 = absent, 1 = focally increased between lobules, 2 = diffusely increased between lobules, and 3 = acini disrupted and separated; (2) inflammatory cell infiltration: 0 = absent, 1 = rare or around ductal margins, 2 = in the parenchyma (<50% of the lobules), and 3 = in the parenchyma (>50% of the lobules); (3) necrosis: 0 = absent, 1 = architectural changes, 2 = pycnotic nuclei, 3 = focal necrosis (<10% of the parenchyma), and 4 = diffuse parenchymal necrosis (>10% of the parenchyma). Histological score was as follows: sum of edema, inflammatory cell infiltration, and acinar necrosis scores.

### 2.8. Statistical Analysis

Data are expressed as mean ± SD. The data was processed by the statistical analysis software SPSS version 16.0 (SPSS Inc., Chicago, IL, USA). Statistical analysis was carried out by one-way analysis of variance (ANOVA) followed by Tukey's multiple comparison test. Nonparametric data were analyzed by Mann-Whitney *U* test. *P* value = 0.05 was statistically significant.

## 3. Result

### 3.1. The Effect of Q10 on Serum Amylase and Lipases

The mean serum amylase level of sham group was significantly higher than the one of control group (172.7 ± 2.82 versus 125.37 ± 2.45; *P* < 0.001). In the E1 and E2 groups, treatment with Q10 caused amylase level to revert to basal levels (158.04 ± 0.98 and 143.58 ± 1.22, resp.). Compared to the sham group, amylase level decreased in both Q10 treated groups (*P* = 0.001). In addition, serum amylase decreased with increasing dosages of Q10 in Q10 treated groups (*P* < 0.001).

Serum lipase levels in the sham group (105 ± 1.39) and treatment groups (E1: 77.04  ±  0.8; E2: 57.95  ±  0.9) were higher than the control group (43.7 ± 0.8; *P* < 0.001). The increase in lipase was markedly reduced in rats treated with Q10 and those groups have the lowest increase in lipase levels. Between two treatment groups, the E2 group performed statistically best in preventing the increase in the lipase levels (*P* < 0.001).

### 3.2. The Effect of Q10 on the Serum Cytokines

**Figure 1:**
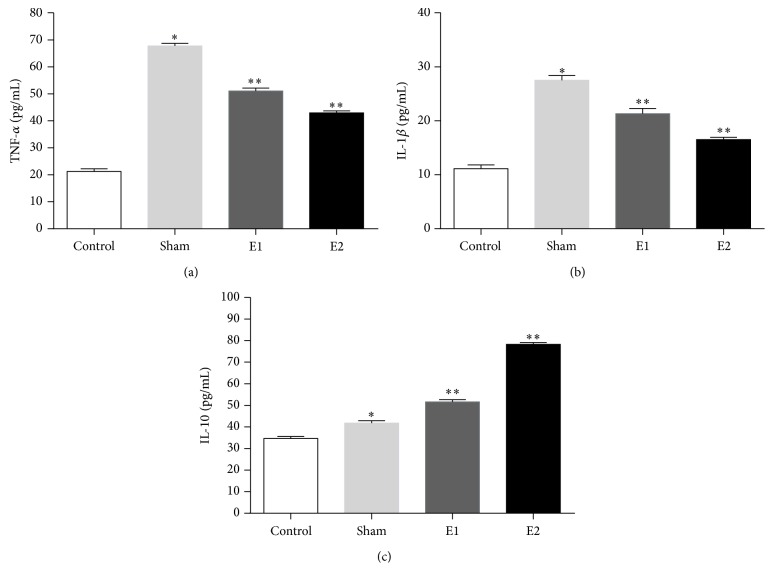
Effects of Q10 on the pro- and anti-inflammatory serum cytokines levels in rats. The contents of serum TNF-*α* (a), IL-1*β* (b), and IL-10 (c) of rats were measured using ELISA kits as described in Materials and Methods. Values are expressed as mean ± SD, *n* = 8. ^*∗*^ *P* < 0.05, versus control group; ^*∗∗*^ *P* < 0.05, Q10 treated groups versus sham group.

TNF-*α* and IL-1*β* levels in control group were found to be significantly lower than in sham group (*P* = 0.024 and *P* = 0.01, resp.). In addition, a significant difference was detected between sham group and Q10 treated groups in terms of TNF-*α* and IL-1*β* levels (Figure 1). Regarding Figure 1, compared to the control group, treatment of rats with Q10 significantly increased IL-10 level in E1 and E2 groups.

### 3.3. The Effect of Q10 on the Oxidative Stress in the Pancreatic Tissue

MDA and MPO levels in the pancreases of sham group were higher than those of the control group, while SOD and GSH levels were significantly lower than those of the control group (Table 1). As showed in Table 1, MDA and MPO levels were significantly increased in the pancreatitis rats, whereas the activities of antioxidant enzymes, such as SOD and GSH, were decreased. However, treatment of rats with Q10 effectively decreased MDA and MPO levels and increased antioxidant enzymes activities in E1 and E2 groups (*P* = 0.005).

### 3.4. Evaluation of Histological Damage in the Pancreatic Tissue

**Figure 2:**
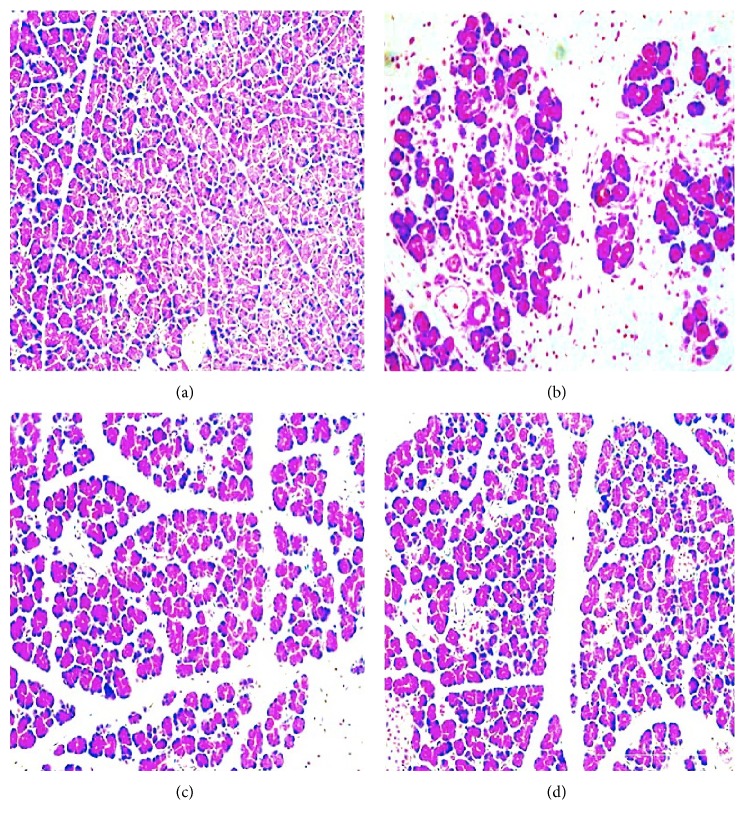
Pictures of pancreatic tissue sections in rats (hematoxylin and eosin, magnification ×200). (a) normal morphology, control group; (b) sham group is characterized by interstitial edema, inflammatory cell infiltration, and acinar cell; (c) and (d) treatment with Q10 (E1 and E2 groups, resp.) resulted in lower interstitial edema, less inflammatory cell infiltration, and alleviated acinar cell necrosis.

**Figure 3:**
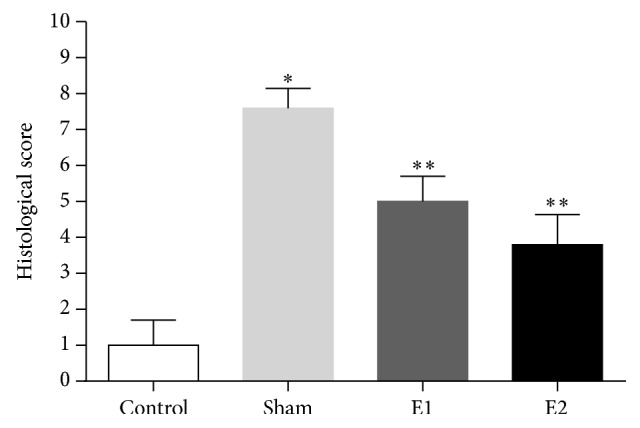
Total histopathological scores in pancreatic tissues of the rats. Data are expressed as mean ± SD (^*∗*^ *P* < 0.001 versus control group; ^*∗∗*^ *P* < 0.05 versus sham group).

Histological examination of pancreas sectioned from L-arginine treated rats revealed tissue damage characterized by edema, inflammatory cell infiltrates, and acinar cell necrosis. Pancreatic edema, inflammation, and necrosis in the sham group were significantly higher than in the control and treatment groups (Figure 2). When assessing the groups in terms of their total histopathological scores, it was observed that the sham group received the highest score, the control group received the lowest score, and the scores of treatment groups were lower than the sham group. Mean histopathological scores are demonstrated in Figure 3.

## 4. Discussion

As current treatment strategies of AP are mainly limited to supportive care, more effective therapeutic options should be developed for better management of this disorder. Thus, in this study we evaluated the effects of Q10, a ubiquitous radical-scavenging antioxidant present in most eukaryotic cells, on a rodent AP model induced by L-arginine. Our data signified that the pharmacological activation of Q10 in acinar cells can result in not only a remarkable reinforcement of antioxidant defense system but also a significant reduction of inflammatory mediators. Previously, many studies have indicated the role of impaired Q10 bioavailability or biosynthesis in several diseases and also evidently demonstrated favorable outcomes of Q10 supplementation in various disorders and deficiencies [20]. Q10 preventive role on ROS production is mainly explained by its actions as a mitochondrial electron carrier that regulates electron acceptance from complex I and II and also an activator of mitochondrial uncoupling proteins that hampers free radical accumulation. Further, inhibition of NF-kappa B by Q10 lowers bcl-2 level which leads to mitochondrial membrane integrity, a mechanism that blocks oxidative-induced apoptosis by hindering the release and activation of degenerative proapoptotic molecules including cytochrome c and caspases 3 and 9 [21, 22]. Although ROS modulate pivotal signaling pathways of redox reactions, cellular environment cannot sustain exorbitant states of oxidative stress. Thus, a combination of physical and chemical barriers including repair enzymes and antioxidant systems is initiated to neutralize ROS. However, tissue damage might emanate from disturbance in redox balance as a result of excessive ROS production [23]. Disruption of acinar cells in AP precipitates pancreatic enzyme leakage, recruitment of inflammatory mediators, and finally ROS aggravation [24].

In the present study, results are in favor of significant protective effect of Q10 on rising of serum amylase and lipase activity, both dependent on treatment dosage. Serum lipase or amylase activities, most commonly used biomarkers in AP, are at least tripled compared to upper limit of normal; however their serum levels may not be dependent on pancreatitis severity [1]. Besides, amylase rise is detected up to 3–5 days, while lipase level stays high for 8–14 days and is therefore more sensitive for diagnosis in delayed presentation [25].

In addition, our findings demonstrated decreased proinflammatory TNF-*α* and IL-1*β* cytokines in treatment groups, and their levels were closer to normal with higher Q10 dosage. IL-1*β* and TNF-*α* are the major and primary proinflammatory mediators and are accountable for all other subsequent systemic complications [6]. Activation of granulocytes and other proinflammatory mediators is regulated by TNF-*α*, which activates intracellular protease (trypsinogen) and thus cellular necrosis [26]. IL-1 plays an important detrimental role in AP, since it is noted as the main mediator in sterile necrosis and local and systemic tissue destruction, with 82% accuracy in prediction of severity [6]. On the other hand, Q10 assisted in boosting of anti-inflammatory cytokine, IL-10, which happened to be consistent with greater dosage of Q10 as well. IL-10 is produced multisystemically, like TNF-*α*, and serves a protective role by controlling production of TNF-*α* [27, 28].

Peroxidation of membrane lipids by ROS releases toxic byproducts such as MDA [29], which in turn leads to activation of complement cascade, other cytokines, and SIRS as a final consequence [8]. MDA is directly associated with tissue injury and organ failure in AP [30]. For this reason and also for its early peak after 3–5 hours going back to normal after 12 hours [7], MDA is considered as a marker of AP severity in early stages [8]. In addition, MPO, another lipid peroxidation byproduct, shows increased plasma level in severe AP [30]. The antioxidant defense consists of SOD, catalases, and GSH [8]. Although SOD prevents lipid peroxidation and has a protective role in tissue necrosis, it leaves tissue edema and inflammatory responses unaffected [7]. In AP, SOD decreases, however without significant correlation with severity [8]. Furthermore, reduced glutathione in pancreas is suggestive of oxidative stress at tissue as well as systemic levels in AP [30]. In our study, the pattern of changes in oxidative stress markers was also in agreement with the previously claimed protective effect of Q10. Both MDA and MPO levels declined according to dosage increase, contrary to SOD and GSH, which elevated with increased dosage. Correspondingly, tissue morphology and structure were also better saved with Q10, especially with its higher dosage.

Attempts to counteract the aforementioned complications of AP by exploiting various antioxidants have provided mixed results. To the best of our knowledge, there is no similar experiment, which has dealt with impact of Q10 on AP. Herein we shortly review previous paper on various antioxidants.

Evaluation of anti-inflammatory and antioxidative effects of lycopene on severe AP in both in vivo and in vitro models by Lv et al. [31] revealed alleviation of pathological changes by lycopene pretreatment including protection against necrosis and apoptosis in acinar cells which was maintained by relieving the mitochondrial and endoplasmic stress, decreased levels of TNF-*α*, serum amylase, C-reactive protein, MDA and lipid peroxidation, and also heightened SOD state. Lycopene beneficial effects on maintenance of cellular homeostasis could be exerted through prevention of JNK pathway phosphorylation [31].

Nuclear factor-erythroid-2-related factor (Nrf2), a regulator of detoxifying molecules and cellular antioxidants, can be activated by dh404. In a study by Robles et al., treatment with synthetic triterpenoid RTA dh404 (CDDO-dhTFEA) in rat pancreas resulted in significant prevention of acinar architecture damage, reduced inflammatory cell infiltration, perilobar edema, and necrosis as well as decreased amylase and MDA, which was compatible with our study. Besides, elevated antioxidant and lower inflammatory mediators' expression with pronounced cellular viability against oxidative stress was observed [32].

In an investigation of AP induced liver injury in rats via antioxidant response, Bakır et al. used carvacrol, the essential oil of* Origanum vulgare*, which possesses a variety of biological and pharmacological properties. Authors found that carvacrol decreased pancreatitis-induced MDA levels as well as AST, ALT, and LDH. Moreover, SOD, CAT, and GSH activities in the treated group were higher. Besides, carvacrol could also alleviate AP induced liver necrosis, coagulation, and inflammation. They concluded that carvacrol could be a safe and potent new drug candidate for treating AP through its antioxidative mechanism of action especially for the treatment of hepatic related disorders [33].

Considering that in previous animal models of AP N-acetylcysteine could decrease the severity of disease, Milewski et al. studied treatment with N-acetylcysteine for AP as one of the most severe and common complications in postendoscopic retrograde cholangiopancreatography (ERCP) patients. However, N-acetylcysteine failed to show substantial preventive effects in such cases [34].

A study in Germany on patients with AP yielded supportive results in favor of advantage of selenium therapy in terms of decreased morbidity and mortality and the number of necessary operations.

Nonetheless, as administration of one or few exogenous compounds cannot compensate for complete natural antioxidant defense system which consists of numerous detoxifying enzymes, therefore, the complex endogenous antioxidant system requires more than only a few aiding compounds to be boosted [35].

Despite fundamental support for the basic role of ROS influx and inflammatory cascades mediated by oxidative stress in AP, currently firm clinical verification regarding clear potential of antioxidant supplementation of AP is still lacking. As several different factors might be involved in induction of AP, which ultimately result in diverse physiologic complications, treatment with combination modalities can be of higher value in clinical settings in comparison to single agent approaches [36].

## 5. Conclusion

Based upon the results of the present study, administration of coenzyme Q10 diminished inflammation by lowering inflammatory mediators and oxidative stress by escalating expression of crucial antioxidant enzymes in experimental AP model. Hence, Q10 seems to be a promising antioxidant with significant therapeutic effects in amelioration of histologic damage via modulation of cytokines and oxidative markers and reduction in plasma levels of pancreatic enzymes. Further studies are necessary to determine the clinical efficacy and detailed mechanisms underlying the beneficial properties of Q10 which might also prove to be targets of other antioxidants with future therapeutic potentials.

## Figures and Tables

**Table 1:** Pancreatic tissue MDA, GSH levels, and SOD activity according to the groups. Data presented as mean ± SD.

	MDA (nmol/g)	MPO (U/mg)	SOD (U/mg)	GSH (*µ*mol/g)
Control	24.62 ± 0.87	0.10	70.37 ± 1.40	0.59 ± 0.10
Sham	62.43 ± 1.45^a^	0.30 ± 0.03^a^	49.68 ± 1.06^a^	1.87 ± 0.18^a^
E1	41.31 ± 0.84^a,b^	0.21 ± 0.01^a,b^	145.56 ± 1.80^a,b^	3.49 ± 0.10^a,b^
E2	34.37 ± 0.95^a,b^	0.13 ± 0.01^a,b^	167.31 ± 1.43^a,b^	4.85 ± 0.22^a,b^

^a^ *P* < 0.05, versus *control group*.

^b^ *P* < 0.01, Q10 treated groups versus *sham group*.
